# Stability of serrated gold coil markers in prostate localization†

**DOI:** 10.1120/jacmp.v12i3.3453

**Published:** 2011-04-18

**Authors:** Larry L. Gates, David J. Gladstone, Mohit S. Kasibhatla, John F. Marshall, John D. Seigne, Eugen Hug, Alan C. Hartford

**Affiliations:** ^1^ Department of Medicine Radiation Oncology Section, Dartmouth‐Hitchcock Medical Center Lebanon NH USA; ^2^ Roger Williams Hospital, 21st Century Oncology Providence RI USA; ^3^ Paul Scherrer Institut Villigen Switzerland

**Keywords:** coils, stability, prostate, fiducial marker, positioning, radiation therapy

## Abstract

We investigated the stability of serrated gold coils (Visicoil) implanted within the prostate glands of patients undergoing definitive external beam radiotherapy for prostate cancer. Radiopaque Visicoils of diameter 0.75 mm and median length 3 cm (range 2–4 cm) were implanted, one into each lobe of the prostate glands of 30 patients planned for external beam treatment. The coils were visualized on CT simulation and again after 25 fractions of treatment (5WK). Data from 30 patients were studied, of whom 19 also received androgen ablation therapy. The average change in the distance between the two coils over five weeks of treatment was 0.8 mm (± 0.6 mm), with a maximum of 2.5 mm in one patient. Average residual errors (standard deviations) for the positions of individual coil segments after five weeks of therapy were only 0.7 mm LAT, 0.6 mm AP, and 0.4 mm SI. The average change in distance between the coils over five weeks compared favorably with published data regarding marker seed stability. Overall, less than a 2 mm margin (i.e., 2 standard deviations) would adequately compensate for positioning uncertainty of the coils in more than 95% of cases.

PACS number: 87.55.kh

## I. INTRODUCTION

Current regimens of highly conformal dose constraints with intensity‐modulated radiation therapy (IMRT) and dose escalation demand precision in patient setup and localization of the prostate gland. Many investigators have shown that the movement of the prostate is common and highly varied in magnitude and direction.^(^ ^1^ ^–^ ^5^ ^)^ Motion of the prostate both between and during fractions of external beam radiotherapy (EBRT) is well documented,^(^ ^1^ ^,^ ^4^ ^–^ ^6^ ^)^ as organ motion due to rectal and bladder filling makes the prostate a dynamic target.^(^ ^4^ ^,^ ^5^ ^,^ ^7^ ^)^ However, traditional portal images are often used to match to the pelvic bones, which does not guarantee that the prostate itself is on target as the prostate moves independently from the pelvic bones.

Fiducial markers within the prostate gland are often used for image‐guided radiotherapy of prostate cancer.^(^ ^1^ ^–^ ^3^ ^)^ As the prostate gland is not visible on portal images, implanted fiducials in the gland allow portal image matching to these markers, resulting in the higher accuracy necessary to gain the full therapeutic advantage of highly conformal treatment plans.

Gold seed markers are the most common form of radiopaque fiducials. A number of articles have analyzed setup errors and/or prostate motion using internal seed markers matched on portal images^(^ ^1^ ^,^ ^8^ ^–^ ^10^ ^)^ or CT scans;^(^ ^2^ ^,^ ^11^ ^,^ ^12^ ^)^ however, these studies often assumed the seeds were static within the prostate. At the extreme, reports^(^ ^13^ ^,^ ^14^ ^)^ have described migration of implanted radioactive brachytherapy seeds out of the prostate capsule and through the vasculature, but investigators have also studied the more limited migration of fiducial markers within the prostate gland itself. Van der Heide et al.^(15)^ evaluated the absolute distance between gold markers for each fraction of radiotherapy using megavoltage portal images. This study compared these distances to those found on the planning CT scan, and demonstrated a gradual decrease in distance over the course of radiotherapy, ending with an average decrease of 0.9 mm after 35 fractions. A study by Poggi et al.^(16)^ observed that the average migration of all seeds in a nine‐patient cohort was 1.2±0.2 mm, but the largest measured change in intraseed spacing was 6.6 mm; a seed was lost in one patient. Kitamura et al.^(6)^ used a 2 mm diameter gold sphere as a single fiducial marker. They measured its migration through the prostate by comparing its position on sequential CT scans relative to the center of mass of the contoured prostate, demonstrating an average absolute migration of 3.0±3.4 mm, with the greatest motion in the craniocaudal dimension. Litzenberg et al.^(3)^ studied the relative positions of three radiopaque prostate markers obtained from the planning CT and compared them to their positions measured from daily orthogonal radiographs. Over the course of 374 fractions evaluated in 10 patients, there were changes in distances between seed positions with the standard deviations of these distances varying from 0.69 to 1.68 mm.

We investigated an alternative fiducial marker system – Visicoil (IBA Advanced Radio‐ Therapy, Fernandina Beach, FL), a serrated gold coil implanted within the prostate glands of patients undergoing definitive external beam radiotherapy. Visicoil is a 0.75 mm diameter gold coil, available in lengths of 1 to 6 cm. Coiled wire markers, as opposed to conventional cylinder shaped seeds, may have less tendency to diffuse and migrate through tissue. The coil's serrations have demonstrated excellent stability in animal models (unpublished data). The purpose of this study is to characterize the degree of motion of these serrated gold coil fiducial markers during prostate external beam radiotherapy in the clinic.

## II. MATERIALS AND METHODS

### A. Subjects

With the introduction into our clinical practice of gold coil fiducial markers for prostate localization, we routinely repeated a planning CT scan at five weeks into radiation therapy to assess whether there was evidence of a significant shift of coil position prior to their utilization as fiducial markers for the boost portion of the prostate treatment. For this retrospective review, we analyzed the first 30 consecutive patients who were implanted with the coils. Of these, a total of 19 patients judged to have higher risk of extracapsular extension or nodal involvement received neoadjuvant and concurrent androgen deprivation therapies, consisting of Casodex, 50 mg daily, along with Lupron depot injections, which extended over the course of the patients' radiation treatments. The mean age of the patients was 71±6 yrs.

Approximately one week prior to treatment simulation, a coil marker was placed under ultrasound guidance, into each lobe of the prostate gland. Patients were treated with 6–10 MV photon beams using IMRT, planned to deliver 1.8 Gy fractions over a course of 42–44 treatment days, with final total doses at 75.6–79.2 Gy to the prostate gland.

**Figure 1:**
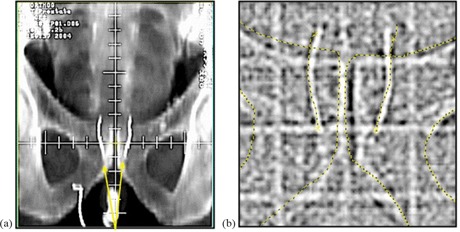
Gold coils implanted in the prostate are shown on a DRR (a) and on an MV portal image (b). Image matching structures obtained from the DRR are superimposed on the EPID to target the coils, rather than the bony anatomy.

The first five weeks (45.0 Gy) of treatment targeted a large PTV that included the lymph nodes, and image matching to the bony anatomy was used. After this, a different PTV that coned down to the prostate was used, and the coil markers were employed to target the prostate directly using the Varian VARiS Vision system (Varian Medical Systems, Palo Alto, CA) along with the electronic portal imaging device (EPID). An example of the image matching using digitally reconstructed radiographs (DRR) with the coil position detected on the EPID is shown in Fig. 1. Computer algorithms then determined relative table shifts, thereby positioning the

planned beams relative to the coil‐defined prostate target. The MUs from the MV portal images were accounted for in the treatment planning process. The contrast of the image in Fig. 1 from the EPID adequately shows the outline of the coils; however, a kV imaging system provides better contrast and also reduces patient dose.

### B. Coil implantation

Radiopaque gold coil markers of diameter 0.75 mm and median length 3 cm (range 2–4 cm) were implanted, one into each lobe of the prostate glands, for patients planned for external beam treatment. A transrectal ultrasound probe was used for image guidance during placement of the two coils. Coil placement was accomplished using two standard 18‐gauge brachytherapy needles inserted under local anesthesia through the perineum.

### C. Coil position analysis

CT simulation scans (SIM) were used for localizing the coils before therapy, and a second CT scan performed after 25 fractions of treatment localized the coils again (5WK). The CT slice thickness was either 1 mm or 3 mm, and the in‐plane resolution was 0.92×0.92 mm. Using the Pinnacle planning system (Pinnacle 6.2b, Philips Medical Systems, Fitchburg, WI), the coil coordinates were identified by digitizing five points along its length, evenly spaced, with a point at each end of the coil. The data was reformatted into an axial, coronal, and sagittal view, and was used to carefully place the points manually. The coronal and sagittal views allowed us to place the points consistently along the coil's length and also mitigated the metallic CT artifact that would sometimes appear at the ends of the coils. Using a fusion algorithm that minimized summed residual errors in all three dimensions for the ten points mapped between SIM and the 5WK scan, the specified points for the SIM scan were mapped to the points for the 5WK scan. This method allowed us to determine the magnitude and direction of changes in relative coil positions between the two scans.

Following image fusion, for each coil the positions of its five points at the time of simulation, $\rho_{\text{SIM},i}$ (where i=1…5) and at five weeks, $\rho_{5\text{WK},i}$, were mapped into a coordinate system (x,y,z). The difference of these vectors (i.e., the shift in relative coil position between scans), the positional vector shift, $\Delta \rho_{i}$, was computed for each point *i* on the coils. Next, the magnitude, $\Delta \rho_{i}$ was computed. Also the tip translation, **w**, of each coil was assessed by defining it as the motion of the coil's top point relative to its bottom point, or as $w=\Delta \rho_{1}-\Delta \rho_{5}$. The magnitude of the tip translation, *w*, was computed and the average *w* across all coils was calculated.

Perceived tip translation could result from either position uncertainty due to imaging (partial volume averaging), or from possible changes in the shape or position of the individual coil.

To determine the change in relative position between the right and left coils, the position of each coil's center was first determined by an average over the coil's five points, $\bar{\rho }=\frac{1}{5}\sum \rho_{i}$. Then, the distance vectors between the right and left coils at simulation and at five weeks are $\Delta {\bar{\rho }}_{\mathrm{sim}}={\bar{\rho }}_{\mathrm{sim},\text{right}}-{\bar{\rho }}_{\mathrm{sim},\text{left}}$, and $\Delta {\bar{\rho }}_{5\text{WK}}={\bar{\rho }}_{5\text{WK},\text{right}}-{\bar{\rho }}_{5\text{WK},\text{left}}$. Next, the difference in the magnitude of these vectors, $\Delta t=∥\Delta {\bar{\rho }}_{5\text{WK}}|-|\Delta {\bar{\rho }}_{\mathrm{sim}}∥$, gives the magnitude of changes in the distance between the right and left coils that resulted between the time of the SIM and the 5WK CT scans.

Standard deviations for the point positions along each coil were calculated for the x‐, y‐, and z‐axes. For each matched pair of points $(\rho_{\text{SIM},i}$ and $\rho_{5\text{WK},i}$), the residual differences in position, $\Delta \rho_{i}$, were calculated in (x,y,z) space. For each of the three axes (x, y, z), the standard deviations for a specific patient's ten matched pairs (i=1,…,5 for the right coil; i=6,…,10 for the left coil) were then calculated,

(1) $$\sigma (\Delta \rho_{i})=\sqrt{\frac{\sum_{i=1}^{10}{(\vec{\Delta \rho_{i}}-\vec{\Delta \rho })}^{2}}{9}}$$

yielding an estimate of the degree of motion over five weeks of radiation treatment of any one point away from its initial position in that dimension for that particular patient. These standard deviations were then compared across all patients, yielding estimates for coil position uncertainty in the lateral (LAT), anterior–posterior (AP) and superior–inferior (SI) dimensions.

## III. RESULTS

Comparison of the top and bottom points of each coil demonstrated some degree of tip translation, *w*, which varied from 0.3 mm up through 5.0 mm, with the largest value seen in one patient who had the tip of a coil resting outside of the prostate gland. All other coils appeared anchored within prostate tissue. The uncertainty in these values is influenced in part by CT scan volume averaging.

**Figure 2:**
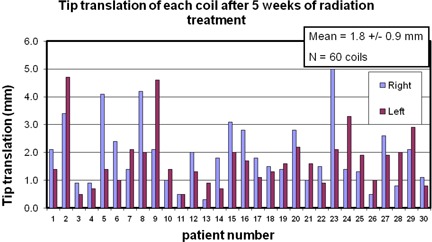
The tip translation, *w*, of each coil for each patient. We defined *w* as the motion of the coil's top point relative to its bottom point. The mean across all 60 coils is shown in the insert.

Across all 60 coils (30 patients, each with two coils), the average measured *w* was found to be 1.8 mm (± 0.9 mm), as reported in Fig. 2.

**Figure 3:**
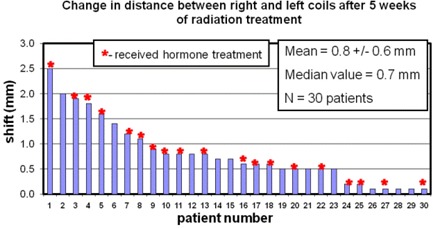
The stability of the coils over five weeks of radiation therapy. For each patient, the relative change in distance between the two coils is plotted. The patient numbers were chosen in order of decreasing observed shift. The patient numbers are consistent in all the figures. An asterisk at the top of a bar indicates that this patient received hormone treatment. The mean shift across all patients is shown in the insert.

**Figure 4:**
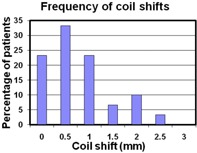
A histogram showing the frequency of coil shifts observed in our cohort. The bin size used was 0.5 mm, centered about the value shown on the x‐axis. These data show that large coil shifts tend to be less frequent than the small shifts, with a median coil shift value of 0.7 mm. The most frequently occurring coil shift is at 0.5 mm (i.e., between 0.25 and 0.75 mm).

For all 30 patients, the average distance between the two coils was 24.9 (± 4.7)mm. However, the average change in the distance between the two coils over five weeks of treatment was only $\bar{\Delta }=0.8\;\text{mm}\;(\pm 0.6\;\text{mm})$, with a maximum of Δt=2.5 mm for one patient. Ordering the data from the highest shift to the lowest, we illustrate in Fig. 3 the change in distance observed for all patients. Only one patient out of 30 had a coil displacement greater than 2 mm, and 83% of patients had shifts less than 1.5 mm. These data can be collapsed to view the regularity of certain coil shifts, as shown in the histogram in Fig. 4. The median value of the coil displacements is 0.7 mm, while the most frequently occurring coil shift is binned at 0.5 mm in Fig. 4, which combines values between 0.25 mm and 0.75 mm.

**Figure 5:**
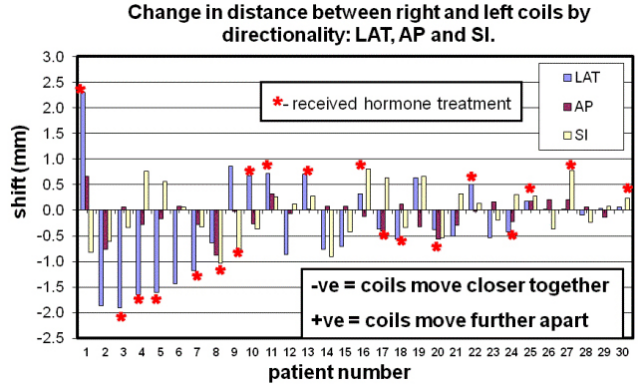
A breakdown of the data shown in Fig. 3. The change in distance in the three directions LAT, AP, and SI are shown to indicate the predominant direction of coil motion. The mean of the absolute shift across all patients for each direction is 0.7±0.6 mm in the LAT, 0.2±0.2 mm in the AP, and 0.5±0.3 mm in the SI direction. Negative distance changes indicate the coils are moving closer together; positive changes indicate they are moving further apart.

While Fig. 3 represents the magnitude of the distance shifts, it is instructive to see the directionality of these changes. Figure 5 shows a breakdown of these data as a plot of the change in the distance between the two coils for the lateral, anterior–posterior and superior–inferior directions. This illustrates the predominant direction driving the magnitude of the changes seen in Fig. 3. The average displacements for each direction were 0.7 mm (± 0.6 mm) in the LAT, 0.2 mm (± 0.2 mm) in the AP, and 0.5 mm (± 0.3 mm) in the SI direction. For those patients with absolute shifts greater or equal to 0.8 mm (patient numbers 1 through 13), Fig. 5 suggests a trend where the LAT directionality appears to dominate the observed shifts. The majority of these patients (8 out of 13) had negative LAT and AP shifts, indicating the coils were moving slightly closer together. For absolute shifts less than 0.8 mm, there does not appear to be a predominant direction for coil motion.

**Figure 6:**
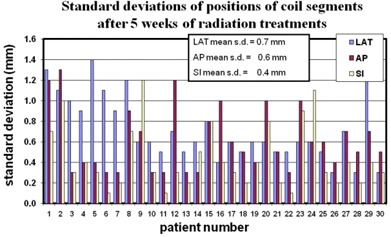
The standard deviations, $\sigma (\Delta \rho_{i})$, of the relative positional change of the 10 digitized points defining the right and left coil between SIM and 5WK scans are shown for each patient. These data give the coil position uncertainty in each direction LAT, AP, and SI. The average across all patients is shown in the insert.

The average residual errors (standard deviations) for the positions of individual coil segments are shown in Fig. 6. The average standard deviations across all patients were 0.7 mm LAT, 0.6 mm AP, and 0.4 mm SI.

## IV. DISCUSSION

This study was designed to measure the relative motion of the coils within the prostate so that coil migration could be assessed. Although uncertainty in mapping or in motion of the tips of the coils yielded measurements of individual coil *w* at greater than 2–3 mm in several cases, the observed stability was excellent, with the average absolute change in distance between the coils over five weeks of treatment at 0.8 mm (± 0.6 mm). Less than 20% of patients had shifts greater than 1.5 mm, and only one out of the 30 patients had a value greater than 2 mm. This intercoil stability suggests the coils anchor well in tissue, with some potential “play” in motion at their tips – likely, in part, reflecting volume averaging between sampled CT slices.

For all parameters tested, differences between patients undergoing and those not undergoing hormonal therapy were not statistically significant. In Fig. 3, those patients who received hormone treatment are identified with an asterisk on the bar graph. There was no observed correlation (p=0.41) of the changes in distance between coils from those without and those with androgen ablation treatment. This may be due to the fact that patients had already received 6–12 weeks of androgen ablation therapy prior to simulation, such that little further measurable volume reduction occurred over the ensuing 6–7 weeks following acquisition of the initial simulation CT scan.

Although hormonal therapy did not prove a significant predictor of changes in magnitude of intercoil distance, Fig. 5 suggests a tendency for coils to move closer together in those patients with larger changes in magnitude of intercoil distance. This may reflect shrinkage of the prostate gland^(17)^ due to either EBRT and/or hormone therapy in patients with larger shift magnitudes, while those with smaller shift magnitudes may be more reflective of random fluctuations and measurement uncertainties. For the larger shift magnitudes, the LAT direction stands out. A possible explanation for this could be the systematic placement of each coil in the right and left lobes of the gland, whereas no attempt was made to offset the coil placement in the other directions.

Overall, the coiled fiducial markers demonstrated their stability within the prostate gland, with minimal motion of the coils relative to one another. This study did not attempt to define the borders of the prostate gland (primarily because no repeat prostate MRI was performed at the five‐week mark, which is the standard we employ for defining the prostate volume) and thus obtain direct measurements of the coil position relative to the prostate center of mass or similar defining landmarks. Nevertheless, we believe it is unlikely that both coils would move within the gland in symmetric fashion, thereby maintaining internal consistency along three dimensions, yet leading to misalignment relative to the prostatic volume. The two coils are flexible and are independent of each other in the prostate; therefore, one would expect migration and deformation forces to affect each coil in a different way.

## V. CONCLUSIONS

Our coil stability data compare favorably with data from other studies of marker seed placement.^(^ ^3^ ^,^ ^6^ ^,^ ^10^ ^,^ ^15^ ^,^ ^16^ ^)^ The coils are 2–4 cm in length with serrations that tend to grip prostatic tissue. This geometry is quite different from conventional small gold seeds, and this may be reflected in our stability data. For example, one study of only nine patients^(16)^ demonstrated average seed migration at 1.2 mm, with changes in interseed distances during a course of therapy as high as 6.6 mm. In the present study of 30 patients, the average coil distance change was only 0.8 mm, with a maximum at 2.5 mm.

The standard deviation of the average change in distance between the two coils across the 30 patients was 0.6 mm (Fig. 3). Average standard deviations for all patients in the lateral, AP, and SI dimensions were 0.7 mm, 0.6 mm, and 0.4 mm, respectively. Therefore at treatment planning, by any of these measures, the safety margin in each dimension needed to account for coil position motion uncertainty during subsequent prostate treatment – encompassing 95% of cases (two standard deviations) – would be considerably less than two millimeters.
